# Management of febrile illness in rural Guinea over a seven-year period: A retrospective study

**DOI:** 10.1371/journal.pgph.0001133

**Published:** 2022-10-12

**Authors:** Karifa Kourouma, Fassou Mathias Grovogui, Alexandre Delamou, Mahamoud Sama Chérif, Brecht Ingelbeen, Abdoul Habib Beavogui, Johan van Griensven, Emmanuel Bottieau

**Affiliations:** 1 Centre National de Formation et de Recherche en Santé Rurale de Maferinyah (CNFRSR) Forécariah, Maferinyah, Guinea; 2 Africa Centre of Excellence for Prevention and Control of Transmissible Diseases (CEA-PCMT), University Gamal Abdel Nasser of Conakry, Conakry, Guinea; 3 Regional Direction of Health, Faranah, Guinea; 4 Department of Public Health, Institute of Tropical Medicine (ITM), Antwerp, Belgium; 5 Department of Clinical Sciences, Institute of Tropical Medicine (ITM), Antwerp, Belgium; PLOS: Public Library of Science, UNITED STATES

## Abstract

**Introduction:**

Febrile illnesses constitute a major clinical challenge in tropical settings. We aimed to assess the frequency, presentation and management of febrile illness at two health facilities in Forécariah, Guinea, with a focus on appropriateness of antibiotic prescription.

**Materials and methods:**

This was a retrospective study conducted in patient files in a health center and a district hospital. Proportions of antibiotic prescription were determined by age group and syndromes as well as appropriateness of antibiotic prescription using the WHO model list (2019).

**Results:**

From 2014 to 2020, 23,583 of 62,185 (38.0%) visits were related to febrile illness. Most patients with fever were female (56.1%) and evaluated at the health center (81.0%). Gastrointestinal (40.6%) and respiratory syndromes (36.8%), and undifferentiated fever (30.0%) were the most common presentations. Malaria was confirmed in 61.3% of the cohort. Overall, the rate of antibiotic prescription was high (14,834/23,583, 62.9%), mostly among patients aged <5 years (5,285/7,566, 69.9%), those with respiratory (7,577/8,684, 87.3%) and gastrointestinal (6,324/9,585, 66.0%) syndromes. Moreover, 7,432/14,465 (51.4%) patients with malaria were also prescribed an antibiotic. Penicillin (42.0%), cotrimoxazole (26.3%) and quinolones (18.7%) were the most frequently prescribed antibiotics. Overall, appropriateness of antibiotic prescription was low (38.3%), and even more so in patients with respiratory (29.1%) and gastrointestinal (25.8%) syndromes.

**Conclusions:**

Febrile illness is a major cause of consultation in rural Guinea. Rate of antibiotic prescription was high, even in confirmed malaria and was often considered inappropriate. There is a pressing need to investigate the etiological spectrum and improve the diagnostic approach of febrile illness in Guinea.

## Introduction

Fever is a common reason of healthcare visit in sub-Saharan Africa [1]. Febrile illnesses constitute a significant diagnostic challenge due to various clinical presentations and countless etiologies [2, 3]. Worldwide, sub-Saharan Africa (SSA) bears the highest burden of infectious diseases with severe febrile illness accounting for 18.4/1,000 hospital visits and more than 16 million hospital admissions (2014) [4]. While the incidence of clinical malaria and related death has dramatically decreased over the past decade [5], other pathogens such as respiratory viruses, arboviruses or bacteria have proportionally increased as etiologies of fever among out- and inpatients in Africa [6]. As well, the emergence of a novel coronavirus in 2020 [7] and the reemergence of the Ebola virus in Guinea (2021) [8] have underlined the importance of collecting accurate epidemiological data. However, factors such poor training of healthcare workers (HCW) and insufficient access to reliable laboratory diagnostic tools in low resource settings, particularly at primary healthcare facilities, hamper the appropriate management of febrile patients. In addition, the deployment of malaria rapid diagnostic tests (RDT) has reduced the overuse of antimalarials but has also led to a paradoxal increase in antibiotic prescription as a default treatment of fever, fueling the spread of antimicrobial resistance [9, 10].

To improve the quality of care for febrile patients, the World Health Organization (WHO) has recommended guidelines based on syndromic approaches such as the integrated management of childhood illnesses (IMCI) or integrated management of adolescent and adult illnesses (IMAI) in settings with limited diagnostic capacity [11, 12]. Yet, evidence shows that SSA countries do not always formally adopt these guidelines, and where they have been deployed, HCW often do not use them [13, 14]. For example, a Tanzanian study assessing the management of febrile children reported lack of consistency between symptoms at admission and diagnosis, with over half of pneumonia diagnoses not meeting the WHO definition [15]. In Malawi, a study found the integrated management of pediatric fever to be suboptimal, with 59% of patients without antibiotic need receiving an antibiotic therapy [14]. Consequently, rates of antibiotic prescription have been reported to be high at primary health facilities in Africa, ranging from 36.7% in Cameroon [16] to 59.9% in Ghana [17]. According to various surveys, penicillin (46%), cotrimoxazole (40%), third-generation cephalosporins (34%) and metronidazole (31%) are the most prescribed antibiotics [16, 18, 19].

In Guinea, fever represents a leading reason for consultation in health facilities [20]. Though the proportion of febrile patients with confirmed malaria has decreased by 14% in the country from 2012 to 2017 [21], the emergence of new epidemics [22] has stressed the importance of adequate diagnosis for appropriate management. Also, information on antibiotic prescription is very limited in the country as only two small hospital-based studies restricted to respiratory tract infections have been published so far to our knowledge [23, 24]. Therefore, we undertook a large retrospective study covering the post-Ebola period 2014–2020 in two health facilities of the rural district of Forécariah, Guinea, to assess (1) the pattern of syndromes at presentation; (2) case management and outcome; and (3) frequency and appropriateness of antibiotic prescription.

## Materials and methods

### Study design and period

This was a retrospective study based on routinely collected data in the rural health district of Forécariah in Guinea during a seven-year period (01/01/2014–31/12/2020).

### General setting

Guinea, located in West Africa, had an estimated population of 12 million inhabitants in 2019 with the majority (70%) living in rural areas [20]. The national public healthcare system is divided into three levels of care, including primary (410 health centers, some of them with inpatient capacity), secondary (seven regional and 38 district hospitals) and tertiary (three national hospitals) [20].

In terms of disease burden, respiratory infections and tuberculosis, neglected tropical diseases, malaria and enteric infections still represent a major health problem in the country [25]. Similarly, preventable childhood diseases are of significant concern as the national extended immunization program coverage was at 24% in 2018 [20].

### Study setting

The rural health district of Forécariah, Basse-Guinée region, had an estimated population of 242,942 inhabitants in 2019 [26], and it comprises one district hospital, one health center with inpatient capacity and eight health centers (including one urban) [27]. The district hospital and the health center with inpatient capacity were the two selected study sites. The selection was based on availability and/or good archiving of data for the study period.

### Study population

We included all patients above one month of age presenting with documented fever (≥38°C, axillary) or reporting a history of fever at any of the selected facilities during the study period.

### General management of febrile patients in Guinea

According to patients’ clinical presentation and the facility level, all patients presenting with fever are first subjected to malaria rapid diagnostic test (RDT), histidine-rich protein-2 (HRP2) based, or blood microscopy to confirm or exclude malaria. After that, additional diagnostic investigations may be implemented based on the diagnostic platform available at the facility. At health center level, malaria RDT may be performed, and HIV testing is offered as part of the mother-to-child HIV transmission program during antenatal care visits. At the health center with inpatient capacity, the district and regional hospitals, further routine laboratory tests such as blood smear for malaria, hepatitis B surface antigen test, full blood count, urine analysis, smear microscopy for tuberculosis and Emmel test for sickle cell disease are usually available. Finally, test determining C-reactive protein (CRP) concentration and blood culture have recently been introduced at two of the three national hospitals (hôpital de l’amitié chinoguinéene and Donka) as advanced diagnostic tools. However, they are not routinely available at peripheral level.

### WHO integrated management of childhood (IMCI), adolescent and adulthood illness (IMAI) guidelines

In response to the limited access to appropriate diagnostic tools in low resource settings, particularly at primary healthcare level, the WHO has recommended a syndromic approach the IMCI and the IMAI to guide healthcare workers in case management, disease prevention and health promotion [11, 12]. These guidelines are considered the state of art management for common illnesses, and they describe the sequence through which care should be provided to a sick patient. The IMCI includes two main parts based on the child’s age (<2 months or ≥2 months to 5 years) because of the differences in clinical presentation and management [12]. For each patient, the Assess, Classify and Identify treatment approach is used. “Assess” stands for checking for “general danger signs” (convulsing or history of convulsion, lethargic, intense vomiting, …). Thereafter, based on present clinical signs, patients are “Classified” as: not severe, moderately severe and very severe. Then, appropriate treatment is “Identified” according to the category in which the patients were classified.

### Source of data and data collection

Data were deidentified and collected using a pretested standardized Excel (2019) sheet and the sources were the emergency unit and the admission (out-inpatients) registers of the two facilities. Data were collected by four research assistants supervised by the principal investigator (KK).

### Study variables

Study variables were sociodemographic characteristics (age, sex, type of facility), clinical symptoms at presentation (documented fever or reported fever, cough, sore throat, rhinorrhea, chest pain, dyspnea, abdominal pain, diarrhea (with or without blood), vomiting, urinary pain, vaginal or ureteral discharge, rash/skin lesions or itch), therapy (antimalaria, antibiotic, antipyretic drugs) and patients’ outcomes (discharged, transferred and death).

In this study, only parasite-based malaria diagnosis was considered confirmed (presence of asexual trophozoites of Plasmodium *spp*. in blood smear or positive malaria RDT). As well, measles, meningitis and tuberculosis were confirmed based on clinical grounds and some additional microscopic analyses since disease presentation is rather specific. We also categorized syndromic presentation according to the following definitions: (1) febrile respiratory syndrome: fever + cough or sore throat or rhinorrhea or chest pain or dyspnea; (2) gastrointestinal syndrome: fever + abdominal pain or diarrhea (with or without blood) or vomiting or nausea; (3) urogenital syndrome: fever + urinary pain or vaginal or ureteral discharge; (4) cutaneous syndrome: fever + rash/skin lesions or itch; and (5) undifferentiated fever: fever without any focal symptom/sign of localization and does not refer to non-acute febrile illness.

### Antibiotic prescription and appropriateness

Prescribed antibiotics in this study were grouped according to the WHO classes and antibiotic groups [28] (S1 File). Also, based on the WHO IMCI/IMAI guidelines, we categorized antibiotic prescription into two groups as follows: (1) “Appropriate antibiotic prescription” if the prescription of antibiotic for the syndromic presentation is always recommended; (2) “Inappropriate antibiotic prescription” if the prescription of antibiotic for the syndromic presentation is never recommended (S2 File).

Finally, we only assessed appropriateness of antibiotic prescription for respiratory and gastrointestinal syndromes as well as for undifferentiated fever (S2 File). We did not assess it for urogenital and cutaneous syndromes as antibiotic are usually considered appropriate or likely appropriate in such cases.

### Statistical analysis

Cleaned data were imported into STATA 16 software (Stata Corporation, College Station, TX, USA) for analysis. Results were summarized using descriptive statistics (proportions, median with interquartile range, IQR). We calculated the overall proportion of different syndromic presentations over the total number of febrile patients in the sample and the overall proportion of major clinical symptoms, confirmed malaria, undifferentiated fever, antipyretic, and antimalarial, antibiotic prescription and for each outcome modality. Overall proportion of confirmed malaria was calculated among each syndromic presentation and a comparison of all results was made by health facility type using a Chi square test and a significance level was set at 5%. As well, overall frequency of antibiotic prescription was determined based on total antibiotic prescription. Then, the rates of antibiotic prescriptions by age groups, syndromic presentation, classes of antibiotics, WHO antibiotic groups and number of prescribed antibiotics were also determined. Finally, the proportion of appropriate antibiotic prescription for selected syndromic presentations (gastrointestinal, respiratory and undifferentiated fever) was determined as described here above.

### Ethical consideration

This study was approved by the National Ethics committee in Research of Guinea (090/CNERS/18) and the Institutional Review Board of the Institute of Tropical Medicine (IRB/AB/AC/058-REF:1490/21). Because this was a retrospective review of routinely data of health facilities, patient consent was not required. Data were de-identified before analysis. Electronic database was kept in a password-protected computer that was only accessible by the principal investigator with regular backups.

## Results

Overall, 62,185 visits took place at the two selected health facilities between 2014 and 2020, of which febrile patients represented 38.0% (23,583/62,185).

Of the 23,583 febrile patients included, females (56.1%) and patients aged ≥15 (49.2%) or <5 years old (32.1%) were predominant. Median age was 14 years (IQR: 3–28). Most visits occurred at the health center with inpatient capacity (81.0%). Cough (33.6%), abdominal pain (26.0%) and vomiting (17.2%) were the most frequently reported symptoms (Table 1), and were more often present in patients seen at the health center with inpatient capacity than in the hospital (p<0.001 for each comparison).

**Table 1:** Sociodemographic characteristics and clinical symptoms of febrile patients (N = 23,583) presenting at the two selected facilities in the rural health district of Forécariah, Guinea, 2014–2020.

Patient characteristics	Total febrile patients (N = 23,583)	Health center with impatient capacity N = 19,097		District hospital N = 4,486		P-value
	N (%)	n	(%)	n	(%)	
**Patient types**						
*Outpatients*	23,109 (98.0)	18,836	(98.6)	4,273	(95.2)	<0.001
*Inpatients*	474 (2.0)	261	(1.4)	213	(4.8)	<0.001
**Sex**						
*Female*	13,234 (56.1)	10,734	(56.2)	2,500	(55.7)	0.561
*Male*	10,349 (43.9)	8,363	(43.8)	1,986	(44.3)	
**Age** *< 5 years*	7,566 (32.1)	6,195	(32.4)	1,371	(30.6)	
*5–14 years*	4,421 (18.8)	3,785	(19.8)	636	(14.2)	<0.001
*≥ 15 years*	11,596 (49.2)	9,117	(47.7)	2,479	(55.3)	
**Age (median, IQR)**	14 (3–28)					
**Clinical symptoms**						
Fever at presentation						
*Reported fever (<38°c)*	16,494 (69.9)	12,974	(67.9)	3,520	(78.5)	<0.001
*Presented with (≥38°c)*	7,089 (30.1)	6,123	(32.1)	966	(21.5)	
Cough	7,930 (33.6)	6,874	(36.0)	1,056	(23.5)	<0.001
Abdominal pain	6,125 (26.0)	4,458	(23.3)	1,667	(37.2)	<0.001
Vomiting	4,048 (17.2)	2,961	(15.5)	1,087	(24.2)	<0.001
Vaginal discharge	1,215 (5.2)	848	(4.4)	369	(8.2)	<0.001
Diarrhea (with or without blood)	1,204 (5.1)	757	(4.0)	447	(10.0)	<0.001
Nausea	680 (2.9)	480	(2.5)	200	(4.5)	<0.001
Rhinorrhea	666 (2.8)	456	(2.4)	210	(4.7)	<0.001
Rash, itching or skin lesions	443 (1.9)	353	(1.9)	90	(2.0)	0.484
Seizure	427 (1.8)	217	(1.1)	210	(4.7)	<0.001
Lethargic	252 (1.1)	40	(0.2)	212	(4.7)	<0.001
Chest pain	165 (0.7)	77	(0.4)	88	(2.0)	<0.001
Dyspnea	136 (0.6)	47	(0.3)	89	(2.0)	<0.001
Sore throat	58 (0.3)	33	(0.2)	25	(0.6)	<0.001
Urinary pain	55 (0.2)	28	(0.2)	27	(0.6)	<0.001
Ureteral discharge	9 (0.0)	7	(0.0)	2	(0.0)	<0.001
Hemoptysis	1 (0.0)	1	(0.0)	0	(0.0)	0.628

IQR = interquartile range. *All results are presented as n/N (%) of fever cases

Table 2 summarizes information about the syndromic presentation, management and outcomes of febrile patients by health facility type. Overall, gastrointestinal (40.6%) and respiratory (36.8%) syndromes were the most common presentation, followed by undifferentiated fever (30.0%). They sometimes presented in combination. All three syndromes were proportionally more often observed in the health center than in the hospital. Of the participants, 61.3% had a confirmed diagnosis of malaria, which was in contrast, much more frequent in the district hospital (accounting for up to 70% of the case load). Of note, malaria was frequently confirmed in all presenting syndromes, particularly in patients with undifferentiated fever (70.4%), gastrointestinal (60.8%) and respiratory (52.5%) syndromes, respectively.

**Table 2:** Syndromic presentation, management and outcomes of febrile patients (N = 23,583) by facility type in the rural health district of Forécariah, Guinea, 2014–2020.

	n/N	(%)		n/N		(%)	n/N	(%)	
**Presenting syndromes**									
**Febrile gastrointestinal syndrome** ^**1**^	9,585/23,583	(40.6)		7,117/19,097		(37.3)	2,468/4,486	(55.0)	<0.001
*Confirmed malaria cases*	*6*,*140/9*,*585*	*(64*.*1)*		*4*,*371/7*,*117*		*(61*.*4)*	*1*,*769/2*,*468*	*(71*.*7)*	*<0*.*001*
**Febrile respiratory syndrome** ^**2**^	8,684/23,583	(36.8)		7,355/19,097		(38.5)	1,329/4,486	(29.6)	<0.001
*Confirmed malaria cases*	*4*,*556/8*,*684*	*(52*.*5)*		*3*,*725/7*,*355*		*(50*.*7)*	*831/1*,*329*	*(62*.*5)*	*<0*.*001*
**Febrile urogenital syndrome** ^**3**^	1,265/23,583	(5.4)		870/19,097		(4.6)	395/4,486	(8.8)	<0.001
*Confirmed malaria cases*	*538/1*,*265*	*(42*.*5)*		*295/870*		*(33*.*9)*	*243/395*	*(61*.*5)*	*<0*.*001*
**Febrile cutaneous syndrome** ^**4**^	443/23,583	(1.9)		353/19,097		(1.8)	90/4,486	(2.0)	0.484
*Confirmed malaria cases*	*165/443*	*(37*.*3)*		*133/353*		*(37*.*7)*	*32/90*	*(35*.*6)*	*0*.*710*
	**Health center with impatient**								
**Variables**	**Total N = 23,583**			**capacity N = 19,097**			**District hospital N = 4,486**		**P- value**
**Undifferentiated fever**	7,077,23,583		(30.0)	6,003/19,097	(31.4)		1,074/4,486	(23,9)	<0.001
*Confirmed malaria cases*	*4*,*982/7*,*077*		*(70*.*4)*	*4*,*201/6*,*003*	*(70*.*0)*		*781/1*,*074*	*(72*.*7)*	*0*.*070*
**Other specific diseases**	412/23,583		(1.7)	223/19,097	(1.2)		189/4,486	(4.2)	<0.001
**Final Diagnosis**									
Confirmed malaria (in total) **	14,465/23,583		(61.3)	11,348/19,097	(59.4)		3,117/4,486	(69.5)	<0.001
**Non-malarial fever**	9,118/23,583		(38.7)	7,749/19,097	(40.6)		1,369/4,486	(30.5)	<0.001
*Febrile gastrointestinal syndrome*	*8*,*797/9*,*118*		*(96*.*5)*	*7*,*513/7*,*749*	*(96*.*9)*		*1*,*284/1*,*369*	*(93*.*8)*	*<0*.*001*
*Febrile respiratory syndrome*	*4*,*128/9*,*118*		*(45*.*3)*	*3*,*630/7*,*749*	*(46*.*8)*		*498/1*,*369*	*(36*.*4)*	*<0*.*001*
*Febrile urogenital syndrome*	*727/9*,*118*		*(8*.*0)*	*575/7*,*749*	*(7*.*4)*		*152/1*,*369*	*(11*.*1)*	*<0*.*001*
**Laboratory tests**									
Widal									
*Positive*	3,032/23,583		(19.9)	1,513/1909	(79.3)		1,519/1,812	(83.8)	<0.001
*Not recorded*	19,862/23,583		(84.2)	17,188/19,097	(90.0)		2,674/4,486	(59.6)	<0.001
HIV testing (positive)	66/608		(10.9)	20/129	(15.5)		46/479	(9.6)	<0.001
**Managements of febrile patients**									
**Antipyretics prescription**	22,464/23,583		(95.3)	18,297/19,097	(95.8)		4,167/4,486	(92.9)	<0.001
**Prescription among confirmed malaria**									
*Did not receive antimalarial*	*1*,*747/14*,*465*		*(12*.*1)*	*1*,*695/11*,*348*	*(14*.*9)*		*52/3*,*117*	*(1*.*7)*	*<0*.*001*
*Were prescribed an antibiotic*	7,432/14,465		(51.4)	4,809/11,348	(42.4)		2,623/3,117	(84.2)	<0.001
**Total antibiotic prescription**	14,834/23,583		(62.9)	11,009/19,097	(57.7)		3,825/4,486	(85.3)	<0.001
**Age groups**									
*< 5 years*	*5*,*285/7566*		*(69*.*9)*	*4*,*106/6*,*195*	*(66*.*3)*		*1*,*179/1*,*371*	*(86*.*0)*	*<0*.*001*
*5–14 years*	*2*,*268/4*,*421*		*(51*.*3)*	*1*,*723/3*,*785*	*(45*.*5)*		*545/636*	*(85*.*7)*	*<0*.*001*
*≥ 15 years*	*7*,*281/11*,*596*		*(62*.*8)*	*5*,*180/9*,*117*	*(56*.*8)*		*2*,*101/2*,*479*	*(84*.*7)*	*<0*.*001*
**Antibiotic prescription rate among syndromic presentation**									
*Febrile urogenital syndrome*	1,148/1,265		(90.8)	800/870	(92.0)		348/395	(88.1)	0.028
*Febrile cutaneous syndrome*	394/443		(88.9)	309/353	(87.5)		85/90	(94.4)	0.062
*Febrile respiratory syndrome*	7,577/8,684		(87.3)	6,393/7,355	(86.9)		1,184/1,329	(89.1)	0.029
*Febrile gastrointestinal syndrome*	6,324/9,585		(66.0)	4,169/7,117	(58.6)		2,155/2,468	(87.3)	<0.001
*Undifferentiated fever*	2,494/7,077		(35.2)	1,654/6,003	(27.6)		840/1,074	(78.2)	<0.001
**Prescribed antibiotics**									
*Penicillin*	6,234/14,834		(42.0)	4,294/11,009	(39.0)		1,940/3,825	(50.7)	<0.001
*Cotrimoxazole*	3,881/14,834		(26.3)	3,670/11,009	(33.3)		211/3,825	(5.5)	<0.001
*Quinolones*	2,778/14,834		(18.7)	1,971/11,009	(17.9)		807/3,825	(21.1)	<0.001
*Third generation cephalosporins*	676/14,834		(4.6)	69/11,009	(0.6)		607/3,825	(15.9)	<0.001
*Macrolides*	566/14,834		(3.8)	395/11,009	(3.6)		171/3,825	(4.5)	0.014
*Imidazole*	552/14,834		(3.7)	353/11,009	(3.2)		199/3,825	(5.2)	<0.001
*Tetracyclines*	393/14,834		(2.6)	360/11,009	(3.3)		33/3,825	(0.9)	<0.001
*Aminoglycosides*	270/14,834		(1.8)	209/11,009	(1.9)		61/3,825	(1.6)	0.226
**WHO antibiotic groups**									
*Access group*	10,918/14,834		(73.6)	8,581/11,009	(78.0)		2,337/3,825	(61.1)	<0.001
*Watch group*	4,081/14,834		(27.5)	2,507/11,009	(22.8)		1,574/3,825	(41.2)	<0.001
**Number prescribed antibiotics**									
*One antibiotic*	14,315/14,834		(96.5)	10,708/11,009	(97.3)		3,607/3,825	(94.3)	<0.001
*Two or more antibiotics*	519/14,834		(3.5)	301/11,009	(2.7)		218/3,825	(5.7)	<0.001
**Outcomes of febrile patients**									
*Discharged*	23,039/23,583		(97.7)	18,633/19,097	(97.6)		4,406/4,486	(98.2)	0.009
*Transferred*	480/23,583		(2.0)	439/19,097	(2.3)		41/4,486	(0.9)	<0.001
*Died*	65/23,583		(0.3)	25/19,097	(0.1)		39/4,486	(0.9)	<0.001

*All results are presented as n/N (%) of fever cases.

**Among confirmed malaria, 65.7% (9,506/14,465) had a positive RDT alone, 27.9% (4,040/14,465) a positive blood smear alone and 7.6% (1,788/23,583) of all febrile patients had no record of RDT or blood smear.

1: Respiratory syndromes were defined as a patient having or reporting fever and/or cough, rhinorrhea, sore throat, chest pain, hemoptysis and dyspnea.

2: Gastrointestinal syndromes were defined as patients having or reporting fever and/or abdominal pain, diarrhea, and vomiting.

3: Urogenital syndromes were defined as patients having or reporting fever and/or urinary pain, vaginal or ureteral discharge.

4: Cutaneous syndromes were defined as patients having or reporting fever and/or skin rash and itch.

5: Other specific diseases number of patients: TB (31), HIV (66), Meningitis (14), Measles (158), Hepatitis (23), Sickle cell disease (30), Otitis (16), Sinusitis (5), Rheumatic fever (86).

Regarding the management of febrile patients, almost all patients were prescribed an antipyretic (95.3%). Surprisingly, about 7,432 (12.1%) of patients with confirmed malaria did not receive antimalarials, and this proportion was significantly higher at the health center as compared to the district hospital (14.9% vs 1.7%) (p<0.001).

Overall, 62.9% (14,834/23,583) of febrile patients were prescribed at least one antibiotic, with the prescription rate being higher at the district hospital (85.3% vs 57.7% in the health center; p<0.001) (Table 2). The frequency of antibiotic prescription was particularly high among febrile patients aged <5 years old (69.9%). Likewise, antibiotic prescription was frequent for all presenting syndromes, particularly patients with respiratory (87.3%) and gastrointestinal (66.0%) syndromes. Of note, 51.4% of patients with confirmed malaria were also prescribed an antibiotic treatment. Compared to the health center, prescription of antibiotic was much more frequent at the district hospital regarding different age groups and among patients with confirmed malaria (Table 2).

Penicillin (42.0%), cotrimoxazole (26.3%) and quinolones (18.7%) were the most prescribed antibiotics (Table 2). Prescribed antibiotics were almost exclusively from the WHO Access (73.6%) and Watch (27.5%) groups. The prescription rate of penicillin (50.7% vs 39.0%), thirdgeneration cephalosporins (15.9% vs 0.6%) and quinolones (21.1% vs 17.9%) was much higher at the district hospital than the health center (p<0.001). In the same line, prescription of antibiotics from the Access group was more frequent at the health center (78.0% vs 61.1%) (p<0.001). In comparison, antibiotic from the WHO Watch group were predominantly prescribed at the district hospital (41.2% vs 22.8%) (p<0.001). Most patients (96.5%) were prescribed a single antibiotic, and this was similar at both facilities. However, prescription of two or more antibiotics was much higher at the district hospital (p<0.001) (Table 2).

Regarding outcomes, 97.7% of the participants were discharged, but 466 (2.0%) were transferred to a higher level of care and death was only documented for 65 (0.3%) of them (Table 2).

The appropriateness of antibiotic prescription is shown in Table 3. Of all prescribed antibiotics, only 38.3% of prescriptions (5,684/14,834) were considered as appropriate (according to the WHO guidelines). Likewise, only 29.1% (2,201/7,577) and 25.8% (1,633/6,324) of antibiotic prescriptions were found appropriate in patients with respiratory and gastrointestinal syndromes, respectively.

**Table 3:** Proportion of appropriate antibiotic prescriptions in each selected category and by facility type in the rural health district of Forécariah, Guinea, 2014–2020.

Variables	Total antibiotic prescription		Health center with impatient capacity		District hospital		P-value
	n/N	(%)	n/N	(%)	n/N	(%)	
Overall appropriate antibiotic prescription	5,684/14,834	(38.3)	4,215/11,009	(38.3)	1,469/3,825	(38.4)	0.897
Appropriate antibiotic prescription among undifferentiated fever	2,463	(98.8)	1,654/1,654	(100.0)	809/840	(96.3)	0.001
Appropriate antibiotic prescription among respiratory syndromes	2,201/7,577	(29.1)	1,883/6,393	(29.5)	318/1,184	(26.9)	0.071
Appropriate antibiotic prescription among gastrointestinal syndromes	1,633/6,324	(25.8)	1,191/4,169	(28.6)	442/2,155	(20.5)	<0.001

## Discussion

To the best of our knowledge, this study is the first to assess patterns of syndromes at presentation, case management, outcome and appropriateness of antibiotic prescription among a large sample of febrile patients in rural Guinea. We found that gastrointestinal (40.6%) and respiratory (36.8%) syndromes predominated in the study population, followed by undifferentiated fever (30.0%). Malaria was confirmed by blood smear or RDT in a large proportion of patients (61.3%). We observed that the overall rate of antibiotic prescription was high (62.9%), in patients aged <5 (69.9%), in all presenting syndromes, particularly those with respiratory (87.3%) and gastrointestinal (66.0%) syndromes. Penicillin, cotrimoxazole and quinolones were the most common prescribed antibiotic with the prescribed antibiotic belonging almost exclusively to the WHO Access (73.6%) and Watch (27.5%) groups. After a critical analysis of the patient files, only 38.3% of the antibiotic prescriptions were considered appropriate with adequacy with WHO guideline being particularly low in patients with respiratory and gastrointestinal syndromes.

This study has several limitations, including the retrospective design, the relatively rigid definition of different syndromes (with the difficulty of classifying mixed symptoms), the absence of longitudinal data about the patient’s outcome (after discharge) and the fact that we were not able to tackle issue of treatment adherence. Although exclusively parasite-based, diagnosis of malaria might have been inflated since some patients might have had another (unrecognized) infection with asymptomatic malaria carriage or RDT might have been falsely positive due to the persistent HRP2 antigenemia [29]. Also, we did not get any information about which type of HCW cared for patients (medical doctor, medical student, nurse, …). Moreover, although based on the WHO guideline, definitions of appropriateness of antibiotic prescription could be somehow arbitrary and could only take into consideration in retrospect what was written in the files (while HCW might have omitted to register important elements of their clinical decision-making). Finally, these findings may not be representative of the whole country, particularly urban settings.

The finding that gastrointestinal and respiratory syndromes, and undifferentiated fever were predominant is in line with previous studies observations in rural Africa [13, 30]. In our study, gastrointestinal symptoms were very frequently reported, at a higher rate than that of most other studies. This may be related to patients and healthcare workers’ interactions like how complaints are reported by patients or captured by HCW. This might also be due to the spectrum of etiologies, which could not be determined in detail. Also, the impact of malaria on the general presentation is difficult to establish as most of the confirmed malaria (65.7%) was histidinerich protein-2 based RDTs. As well, the study showed that the proportion of patients with confirmed malaria who did not receive antimalarials was significantly higher at the health center than at the district hospital. This may be related to several factors, including the poor management of patients’ records (paper-based), and the fact that healthcare workers may forget to fill the register due to workload. Also, HCW would not prescribe antimalarials for patients with confirmed malaria because they suspect other infections than malaria, or antibiotics are often prescribed before the results are made available (and the patient is gone).

We found that rate of antibiotic prescription was high (62.9%), among febrile patients aged <5 years old, and proportionally in all presenting syndromes, particularly in respiratory and gastrointestinal syndromes, and that this prescription was appropriate in a minority of the cases within the two of the three dominant syndromes (gastrointestinal and respiratory). Our results about the high rate of antibiotic prescription are similar to previous studies [31–33] but much higher than that of a systematic review reporting on changes in prescribing patterns from 1995 to 2015 in the WHO African region (46.8%) [34]. The predominance of antibiotic prescription in patients aged <5 years old may be due to the high frequency of respiratory infections among this group and the limited knowledge about the actual epidemiology of fever in the country.

The few studies that investigated in depth the etiologies of febrile illness in sub-Saharan Africa (almost exclusively East Africa) have demonstrated that most respiratory infections are due to viruses in children (like what is observed in Western countries) [33, 35, 36]. Similarly, while most gastrointestinal infections are due to bacteria in the tropics, they are almost always selflimiting and require hydration and symptomatic care. Antibiotics are in fact only recommended in the tiny proportion of patients with dysenteric symptoms [12]. Antibiotic misuse in this group of patients may be partly driven by the erroneous perception among health care workers in African countries, including possibly Guinea that enteric fever is frequent, in relation to the widespread use of the Widal assay, with very poor diagnostic performance [37]. Another worrying observation is the use of antibiotic for (uncomplicated) malaria patients, while usually this is only justified for the subgroup of severe malaria admitted to the hospital, where bacterial co-infection may be of concern.

Our findings showed that penicillin, cotrimoxazole and quinolones were the most prescribed antibiotics at the primary health care level. In contrast, third generation cephalosporins, quinolones and antibiotics belonging to the WHO Watch group were more frequently administered (often in combination) at the hospital. Our results are concurrent with studies from Malawi, Cameroon and Botswana assessing clinical management and prescription practices among febrile patients at primary care level [13, 16, 18]. At the health center level, penicillin and cotrimoxazole are easily accessible in Guinea [38], maybe explaining the general antibiotic overuse for the major clinical scenarios (gastrointestinal and respiratory infections). It is important to note that the retrospective survey could not fully apprehend the higher clinical complexity of hospital cases. Also, the appropriateness of the indication for and type of antibiotic was more challenging to assess. However, our study found that only 38.3% of prescribed antibiotics appeared appropriate. Despite, this low rate is better than those reported by van de Maat et al., and Elfving K et al. (22%) in their studies assessing presumed infections requiring antibiotic prescription in febrile children in rural Tanzania [30, 33], but head-to-head comparisons between settings is not fully adequate. All in all, however, the critical review of patient files strongly suggested that robust antibiotic stewardship should be urgently implemented at both levels of care to prevent antimicrobial resistance through interactive training of healthcare workers, face to face case discussion with mentors and awareness raising among them. There is a pressing need to investigate the etiologies of febrile illness and introduce appropriate diagnostic tests such as point-of-care generic biomarkers to help differentiate bacterial from viral infection particularly at the primary care level, and an evidence-based set of relevant pathogen-specific RDTs.

## Conclusions

Most febrile illnesses at primary care in rural Guinea presented as gastrointestinal and respiratory syndromes or undifferentiated fever, which should be addressed in priority during medical education. Rate of antibiotic prescription was high in most groups of patients and for all presenting syndromes (and even confirmed malaria). In contrast, such prescription often appeared as not justified quantitatively and qualitatively after critical analysis. Finally, there is a pressing need to investigate the etiologies of febrile illness and introduce appropriate diagnostic tests to improve quality of care for febrile patients but also interventions aiming to reduce antibiotic overconsumption.

## Supporting information

S1 File Classes of and WHO antibiotic groups assessed in this study. (DOCX)

S2 File Update of major syndromic presentation along with recommendations about their management according to the WHO IMCI (2014) and IMAI (2004). (DOCX)

## References

[1] Prasad N, Sharples KJ, Murdoch DR, Crump JA. Community prevalence of fever and relationship with malaria among infants and children in low-resource areas. American Journal of Tropical Medicine and Hygiene. 2015;93: 178–180. doi: 10.4269/ajtmh.14-0646 25918207 PMC4497891

[2] Holmes KK, Bertozzi S, Bloom BR, Jha P. Disease Control Priorities, Third Edition: Major Infectious Diseases. Third. Washington, DC: World Bank. 2017. doi: 10.1596/978-1-4648-0524-0 30212102

[3] Prasad N, Murdoch DR, Reyburn H, Crump JA. Etiology of severe febrile illness in low- and middle-income countries: A systematic review. PLoS ONE. Public Library of Science; 2015. p. e0127962. doi: 10.1371/journal.pone.0127962 26126200 PMC4488327

[4] Roddy P, Dalrymple U, Jensen TO, Dittrich S, Rao VB, Pfeffer DA, et al. Quantifying the incidence of severe-febrile-illness hospital admissions in sub-Saharan Africa. PLoS ONE. 2019;14. doi: 10.1371/journal.pone.0220371 31344116 PMC6657909

[5] Steketee RW, Choi M, Linn A, Florey L, Murphy M, Panjabi R. World Malaria Day 2021: Commemorating 15 Years of Contribution by the United States President’s Malaria Initiative. The American Journal of Tropical Medicine and Hygiene. 2021;104: 1955–1959. doi: 10.4269/ajtmh.21-0432 33891560 PMC8176495

[6] Maze MJ, Bassat Q, Feasey NA, Mandomando I, Musicha P, Crump JA. The epidemiology of febrile illness in sub-Saharan Africa: implications for diagnosis and management. Clinical Microbiology and Infection. 2018. pp. 808–814. doi: 10.1016/j.cmi.2018.02.011 29454844 PMC6057815

[7] Zhu N, Zhang D, Wang W, Li X, Yang B, Song J, et al. A Novel Coronavirus from Patients with Pneumonia in China, 2019. New England Journal of Medicine. 2020;382: 727–733. doi: 10.1056/NEJMoa2001017 31978945 PMC7092803

[8] World Health Organization. New Ebola outbreak declared in Guinea. In: Regional Office for Africa [Internet]. 14 Feb 2021 [cited 25 Jun 2021]. Available: https://www.afro.who.int/news/new-ebola-outbreak-declared-guinea

[9] Crump JA, Kirk MD. Estimating the Burden of Febrile Illnesses. PLoS Negl Trop Dis. 2015;9: e0004040. doi: 10.1371/journal.pntd.0004040 26633014 PMC4668833

[10] Reddy EA, Shaw A V, Crump JA. Community-acquired bloodstream infections in Africa: a systematic review and meta-analysis. The Lancet Infectious Diseases. 2010;10: 417–432. doi: 10.1016/S1473-3099(10)70072-4 20510282 PMC3168734

[11] World Health Organization. Integrated management of adolescent and adult illness (IMAI). Geneva; 2002 Sep. Available: https://apps.who.int/iris/bitstream/handle/10665/68055/WHO_CDS_STB_2003.22.pdf?sequence=1&isAllowed=y 26290923

[12] World Health Organization. Integrated Management of Childhood Illness (IMCI)_chart booklet. Geneva; 2014 Jun. Available: https://www.who.int/publications/i/item/9789241506823

[13] Kapito-Tembo A, Mathanga D, Bauleni A, Nyirenda O, Pensulo P, Ali D, et al. Prevalence and clinical management of non-malarial febrile illnesses among outpatients in the era of universal malaria testing in Malawi. American Journal of Tropical Medicine and Hygiene. 2020;103: 887–893. doi: 10.4269/ajtmh.18-0800 32588795 PMC7410417

[14] Krüger C, Heinzel-Gutenbrunner M, Ali M. Adherence to the integrated management of childhood illness guidelines in Namibia, Kenya, Tanzania and Uganda: Evidence from the national service provision assessment surveys. BMC Health Services Research. 2017;17. doi: 10.1186/s12913-017-2781-3 29237494 PMC5729502

[15] Reyburn H, Mwakasungula E, Chonya S, Mtei F, Bygbjerg I, Poulsen A, et al. Clinical assessment and treatment in paediatric wards in the north-east of the United Republic of Tanzania. Bull World Health Organ. 2008;86: 123–139. doi: 10.2471/blt.07.041723 18297168 PMC2647389

[16] Chem ED, Anong DN, Akoachere J-FFKT. Prescribing patterns and associated factors of antibiotic prescription in primary health care facilities of Kumbo East and Kumbo West Health Districts, North West Cameroon. PLoS ONE. 2018;13. doi: 10.1371/journal.pone.0193353 29505584 PMC5837085

[17] Ahiabu MA, Tersbøl BP, Biritwum R, Bygbjerg IC, Magnussen P. A retrospective audit of antibiotic prescriptions in primary health-care facilities in Eastern Region, Ghana. Health Policy and Planning. 2016;31: 250–258. doi: 10.1093/heapol/czv048 26045328 PMC4748131

[18] Mashalla Y, Setlhare V, Massele A, Sepako E, Tiroyakgosi C, Kgatlwane J, et al. Assessment of prescribing practices at the primary healthcare facilities in Botswana with an emphasis on antibiotics: Findings and implications. International Journal of Clinical Practice. 2017;71. doi: 10.1111/ijcp.13042 29178350

[19] Lakoh S, Adekanmbi O, Jiba DF, Deen GF, Gashau W, Sevalie S, et al. Antibiotic use among hospitalized adult patients in a setting with limited laboratory infrastructure in Freetown Sierra Leone, 2017–2018. International Journal of Infectious Diseases. 2020;90: 71–76. doi: 10.1016/j.ijid.2019.10.022 31655112

[20] Institut National de la Statistique. Enquête Démographique et de Santé, EDS 2018. Conakry, Guinea; 2019. Available: http://www.stat-guinee.org/index.php/publicationsins/rapports-d-enquetes/category/86-enquetes-demographiques-et-de-sante-et-enquetesmics?download=168:rapport-d-enquete-demographique-et-de-sante-2018

[21] Institut National de la Statistique. Enquête de prevalence parasitaire du paludisme et de l’anemie- Guinée 2016. Conakry, Guinée; 2017. Available: https://dhsprogram.com/pubs/pdf/FR332/FR332.pdf

[22] Kolie D, Van De Pas R, Fofana TO, Delamou A, Put W Van De, Van Damme W. Guinea’s response to syndemic hotspots. BMJ Global Health. 2021;6: e006550. doi: 10.1136/bmjgh-2021-006550 34607893 PMC8491297

[23] Sow MS, Camara A, Fortes Deguenonvo L, Medila MA, Barry M, Diallo AAS, et al. Evaluation de la prescription des antibiotiques au cours des infections respiratoires basses chez l ‘ adulte au CHU de Conakry. RevCAMES-Série A, Sciences et Médecine. 2012;13: 239–243.

[24] Talassone Bangoura S, Toure A, Sidibé S, Camara A, Sylla D, Keita A-K, et al. Frequency and Determinants of Prescribing Antibiotics for Internal Medicine at Donka National Hospital (Guinea). Central African Journal of Public Health. 2020;6: 1. doi: 10.11648/j.cajph.20200601.11

[25] Institute for Health Metrics and Evaluation. GBD Compare | IHME Viz Hub Guinea. 2019. Available: https://vizhub.healthdata.org/gbd-compare/

[26] Institut National de la Statistique. Recensement general de la population et de l’habitation_Perspectives Démohraphiques. Conakry, Guinea; 2017. Available: http://www.stat-guinee.org/images/Documents/Publications/INS/rapports_enquetes/RGPH3/RGPH3_perspectives_demographiques.pdf

[27] Direction Préfectorale de la Santé (DPS) de Forecariah. Rapport de la situation sanitaire de Forécariah. Forécariah, Guinée; 2018.

[28] World Health Organization. World Health Organization Model List of Essential Medicines. Geneva; 2019. Available: https://apps.who.int/iris/bitstream/handle/10665/325771/WHO-MVP-EMP-IAU2019.06-eng.pdf?ua=1

[29] Hosch S, Yoboue CA, Donfack OT, Guirou EA, Dangy J-P, Mpina M, et al. Analysis of Nucleic Acids Extracted from Rapid Diagnostic Tests Reveals a Significant Proportion of False Positive Test Results Associated with Recent Malaria Treatment. SSRN Electronic Journal. 2021;21: 23. doi: 10.2139/ssrn.3839416 PMC8785039 35073934

[30] Elfving K, Shakely D, Andersson M, Baltzell K, Ali AS, Bachelard M, et al. Acute Uncomplicated Febrile Illness in Children Aged 2–59 months in Zanzibar–Aetiologies, Antibiotic Treatment and Outcome. Lin B, editor. PLOS ONE. 2016;11: e0146054. doi: 10.1371/journal.pone.0146054 26821179 PMC4731140

[31] Mekuria LA, de Wit TFR, Spieker N, Koech R, Nyarango R, Ndwiga S, et al. Analyzing data from the digital healthcare exchange platform for surveillance of antibiotic prescriptions in primary care in urban Kenya: A mixed-methods study. PLoS ONE. 2019;14. doi: 10.1371/journal.pone.0222651 31557170 PMC6762089

[32] Mabiala Babela JR, Ollandzobo Ikobo LC, Mbika Cardorelle A, Moyen G. Évaluation de l’antibiothérapie initiale en milieu pédiatrique au CHU de Brazzaville (Congo). Medecine et Sante Tropicales. 2013;23: 189–192. doi: 10.1684/mst.2013.0173 23797761

[33] van de Maat J, De Santis O, Luwanda L, Tan R, Keitel K. Primary Care Case Management of Febrile Children: Insights From the ePOCT Routine Care Cohort in Dar es Salaam, Tanzania. Frontiers in Pediatrics. 2021;9: 626386. doi: 10.3389/fped.2021.626386 34123960 PMC8192830

[34] Ofori-Asenso R, Brhlikova P, Pollock AM. Prescribing indicators at primary health care centers within the WHO African region: A systematic analysis (1995–2015). BMC Public Health. 2016;16. doi: 10.1186/s12889-016-3428-8 27545670 PMC4993007

[35] D’Acremont V, Kilowoko M, Kyungu E, Philipina S, Sangu W, Kahama-Maro J, et al. Beyond malaria—Causes of fever in outpatient Tanzanian children. New England Journal of Medicine. 2014;370: 809–817. doi: 10.1056/NEJMoa1214482 24571753

[36] Wang H, Zhao J, Xie N, Wang W, Qi R, Hao X, et al. A Prospective Study of Etiological Agents Among Febrile Patients in Sierra Leone. Infectious Diseases and Therapy. 2021. doi: 10.1007/s40121-021-00474-y 34173960 PMC8234757

[37] Deksissa T, Gebremedhin EZ. A cross-sectional study of enteric fever among febrile patients at Ambo hospital: prevalence, risk factors, comparison of Widal test and stool culture and antimicrobials susceptibility pattern of isolates. BMC Infectious Diseases. 2019;19: 288. doi: 10.1186/s12879-019-3917-3 30917795 PMC6437987

[38] Direction Nationale de la Pharmacie et des Laboratoires. Guide Thérapeutique National. Conakry, Guinea; 2013. Available: https://www.who.int/selection_medicines/country_lists/GuineeConakry_STG_2013.pdf?ua=1

