# Supplementary material for: Management of febrile illness in rural Guinea over a seven-year period: A retrospective study
Source: PLOS Glob Public Health. 2022 Oct 12;2(10):e0001133. doi: 10.1371/journal.pgph.0001133 (PMC10021211; doi:10.1371/journal.pgph.0001133)
Supplement: S1 File — (DOCX) [file pgph.0001133.s001.docx]

**S1 File. Classes of and WHO antibiotic groups assessed in this study.**

| **Classes of Antibiotic** | **Antibiotics** |
| --- | --- |
| Penicillin | Amoxicillin, Amoxicillin-clavulanate, Ampicillin, Cloxacillin, Extencillin, Benzathine penicillin |
| Third generation cephalosporin | Ceftriaxone, Cefixime |
| Quinolone | Ciprofloxacin, Norfloxacin |
| Macrolides | Azithromycin, Erythromycin |
| Aminoglycoside | Gentamicin, Kanamycin |
| Sulfonamides | Cotrimoxazole |
| Tetracyclines | Doxycycline, Tetracycline |
| Imidazole | Metronidazole, Tinidazole |
| **WHO antibiotic groups** | |
| Access group | amoxicillin, ampicillin, cloxacillin, benzylpenicillin, amoxicillin + clavulanic acid, gentamicin etc. |
| Watch group | ciprofloxacin, azithromycin, ceftriaxone, vancomycin etc. |
| Reserve group | colistin, ceftazidime + avibactam etc. |
