# Supplementary material for: Management of febrile illness in rural Guinea over a seven-year period: A retrospective study
Source: PLOS Glob Public Health. 2022 Oct 12;2(10):e0001133. doi: 10.1371/journal.pgph.0001133 (PMC10021211; doi:10.1371/journal.pgph.0001133)
Supplement: S2 File — (DOCX) [file pgph.0001133.s002.docx]

**S2 File. Update of major syndromic presentation along with recommendations about their management according to the WHO IMCI (2014) and IMAI (2004).**

| **Syndromes** | **Clinical presentation** |
| --- | --- |
| **Group 1: Appropriate antibiotic prescription (syndromic presentation for which antibiotic are always recommended)** | |
| Respiratory syndromes | Fever (≥38°C) + chest pain, dyspnea, hemoptysis |
| Gastrointestinal syndromes | Fever or reported fever + dysentery or bloody diarrhea |
| Undifferentiated fever | Fever alone + lethargic + patient was transferred, seizures |
| **Group 2: Inappropriate antibiotic prescription (syndromic presentation for which antibiotic are not recommended)** | |
| Respiratory syndromes | Fever (<38°C) + Rhinorrhea and/or sore throat; Fever (<38°C) + cough and/or dyspnea |
| Gastrointestinal syndromes | Diarrhea without blood, vomiting, abdominal pain |
| Undifferentiated fever | Fever or reported alone |
